# Chronic diseases and determinants of community health services utilization among adult residents in southern China: a community-based cross-sectional study

**DOI:** 10.1186/s12889-024-18435-8

**Published:** 2024-03-28

**Authors:** Junwang Gu, Qi Wang, Wei Qiu, Chunmei Wu, Xiaoqiang Qiu

**Affiliations:** 1 Department of Epidemiology and Health Statistics, School of Public Health, Guangxi Medical University, No. 22 Shuangyong Road, 530021 Nanning, Guangxi China; 2 School of Public Health and Health Management, Gannan Medical University, 341000 Ganzhou, Jiangxi China

**Keywords:** Chronic diseases, Community health services, Utilization, Determinants, China

## Abstract

**Background:**

The burden of chronic diseases has become a major public health concern, and high-efficiency use of community health services is essential in combating chronic diseases. This study described the status of chronic diseases in southern China and explored the determinants of health service utilization among adult residents.

**Methods:**

Data were obtained from one part of community survey data from four counties in Ganzhou City, southern China. A multistage, stratified random sampling method was used to conduct a cross-sectional survey between 2018 and 2020. Overall, 7430 valid questionnaires were collected. A lasso-linear regression analysis was performed to explore the determinants of community health service utilization.

**Results:**

According to the study, most participants (44.6%) reported having relatively good health, while 42.1% reported having moderate health. Chronic diseases were reported by 66.9% of the respondents. The three most prevalent self-reported chronic diseases were hypertension (22.6%), hyperlipidemia (5.9%), and diabetes (5.9%). Among residents with chronic diseases, 72.1% had one chronic disease, while the rest had multiple. Only 13.9% of residents frequently utilized community health services, while 18.9% never used them. Additionally, among residents who reported having chronic diseases, 14.1% had never attended community health services. Four categories of factors were the key determinants of community health service utilization: (1) personal characteristics, age, and sex; (2) health-related factors, such as family history, self-reported health conditions, and the number of chronic diseases; (3) community health service characteristics, such as satisfaction with and accessibility to community health services; and (4) knowledge of chronic diseases. Specifically, women tend to utilize healthcare services more frequently than men. Additionally, residents who are advanced in age, have a family history of chronic diseases, suffer from multiple chronic conditions, rate their self-reported health condition as poor, have a better knowledge about chronic diseases, have better accessibility to community health services, and have higher the satisfaction with community health services, tend to utilize them more frequently.

**Conclusions:**

Given the limited healthcare resources, the government should promote the effective utilization of community health facilities as a critical community-based strategy to combat the growing threat of chronic diseases in southern China. The priority measures involve enhancing residents’ access to and satisfaction with community health services and raising awareness of chronic illnesses among older individuals with poor health status.

**Supplementary Information:**

The online version contains supplementary material available at 10.1186/s12889-024-18435-8.

## Background

The disease spectrum of the Chinese population has changed greatly because of socioeconomic developments, advances in medical and health services, and lifestyle changes. Smallpox, sexually transmitted diseases, and many other infections have either been eliminated or decreased in incidence since 1949. However, the overall incidence of infectious diseases increased from 2004, and the rate leveled off after 2009 [1, 2]. In contrast to the traditional infectious diseases that threatened the health of the Chinese populace, chronic diseases represent a contemporary malady. The observational study displayed that the prevalence of chronic diseases in China has risen rapidly, from 17.0% in 1993 to 34.3% in 2018 [3].

Chronic diseases, also known as noncommunicable chronic diseases (NCDs), are a group of diseases caused by a combination of genetic, physiological, environmental, and behavioral factors with an insidious onset, long duration, and a lack of definite etiological evidence [4]. Chronic diseases have emerged as a major public health problem threatening the population’s health and imposing a heavy disease burden. According to the World Health Organization data [4], NCDs kill 41 million people yearly, equivalent to 74% of all deaths globally. Each year, 17 million people die from an NCD before age 70, and 77% of these deaths occur in low- and middle-income countries.

The government organizes community health services to meet the needs of the public and solve the basic health problems of the community as primary health institutions [5]. Developing the community healthcare service system has an obvious positive effect on optimizing the public health service system, achieving equity, controlling the rapid growth in the medical costs of public health services, and improving the population’s health [5, 6]. A study showed that community health centers are associated with a 2% reduction in age-adjusted mortality rates among those aged 50 and above [7]. Currently, community health services focus on chronic diseases among community residents and are a key part of chronic disease management [5, 8].

Over the past decade, China has implemented substantial healthcare reforms to enhance primary healthcare services [5]. This effort has resulted in the establishment of community health facilities and designated primary healthcare providers as “gatekeepers.“ [5] This effort has yielded impressive results, as communities have taken charge of primary healthcare, improving health outcomes and reducing costs for chronic illnesses [9]. In 2009, the Chinese government set aside 15 Yuan (approximately 2.2 USD, at 2009 conversion rates) per person yearly for national public health intervention programs. By 2011, this budget had increased to 25 Yuan (approximately 3.9 USD, at 2011 conversion rates) per person annually [10]. 60% of these funds were designated for township health centers in rural areas to carry out public health interventions, with the remaining 40% allocated to village health stations [10]. Within this budget, 18% was devoted to managing patients with hypertension, and 7% was allotted to managing patients with diabetes [10]. Nowadays, prioritizing a community-focused strategy to address NCDs has been recognized as a highly cost-efficient solution in China.

However, given the growing burden of chronic diseases, health-service needs are not always met [11]. A study of Chinese adults aged 45 years and older revealed that the prevalence of unmet healthcare needs was 13.0%, and it was more concentrated among people living with multiple health conditions and mental health problems [11]. Increasing investment in medical resources does not fully match the growth of health [12]. Two principal causes are probably involved in the current dilemma: inadequate health services and inefficient utilization of health services, seriously limiting the effectiveness of chronic disease management efforts [13, 14]. Although the government has been allocating more resources to health services, health resources are always limited, and improving the efficiency of health service utilization is an issue for government departments to consider [12, 13].

Although there is sufficient literature on the discussion of health service inequities [12, 13, 15], further research is needed to investigate the factors contributing to such disparities in health service utilization. The previous study found that illnesses like diabetes substantially increased service utilization across the healthcare system [16]. A study conducted among homeless adults revealed that those who were 45 years or older, female, diagnosed with a mental or substance use disorder, or had been homeless for more than a year were more likely to use health services frequently [17]. According to the 2008 Korean Longitudinal Study of Ageing, health-related needs and income were significant predictors of Korean elders’ use of physician services [18]. Among Pakistani immigrants in Maryland, USA, individuals with higher income, a longer stay duration, higher education level, and male gender utilized health services more frequently [19]. In China, data from the 2015 Migrant Dynamics Monitoring Survey found that age, marital status, income, years of residence, migration range, reasons for migration, friend network size, health insurance, local health insurance status, and chronic disease status were linked to health services use among elderly migrants [20]. However, prior research investigating the factors affecting healthcare utilization has focused on specific diseases or certain groups of people, limiting their findings’ generalizability. Systematic investigations into factors affecting utilization of community healthcare services in China still need to be completed.

Ganzhou City, located in the southern region of China and the largest city in Jiangxi Province, boasts a moderate economy. Despite its unique position as a “core city” in inland provinces, Ganzhou does not benefit from the economic radiation of developed cities around the Yangtze River Delta region, nor is it connected to the developed Pearl River Delta region, resulting in a relative lag in economic development and primary healthcare service system. Nevertheless, Ganzhou has made significant efforts to reform its healthcare sector in recent years, with the construction of close medical consortiums launched across the city in 2019, ushering in a new stage in the practice of the health service system. The community health services offered in the region are a good representation of those provided in Southern China. However, Considering the lack of existing relevant research, there is still a need to conduct a thorough study on the current characteristics of community health services utilization and the related determinants in Southern China [21]. Thus, the current study aimed to describe the current situation of chronic diseases among adult residents in communities in Southern China, further explore the key determinants affecting the utilization of community health services, and provide a theoretical basis for community management of chronic diseases.

## Methods

### Design and participants

Data were obtained from one part of the community survey from four counties in Ganzhou City, southern China. A multistage, stratified random sampling method was used to conduct a cross-sectional survey between 2018 and 2020. Permanent residents aged 18 years and older who had settled in the area for 6 months or more were selected. A stratified sample of community residents in the four selected counties was collected at different time stages. Our research group conceived a survey project concerning the official community questionnaires, implemented after discussion and validation with relevant professional experts and the Centers for Disease Control and Prevention (CDC) staff. The survey was conducted under the guidance of the related county Health Commission and organized by the County-level CDC in conjunction with the township health centers.

### Sampling methodology

The sampling methodology involved three stages. During stage one, the townships in each district were divided into three strata based on their economic status. Two townships were then randomly selected from each stratum. In stage two, one or two administrative villages or resident committees were randomly chosen from the selected townships. Finally, stage three randomly chose one or two villagers or resident groups from the selected administrative villages or resident committees. The research participants were then randomly selected from the chosen villagers or resident groups for the investigation.

For details regarding the population under study, the employed sampling method, and the sample size calculation, please refer to the relevant description in our previously published paper [21].

### Instruments

This study collected respondents’ personal information, chronic diseases, and determinants of community health service utilization using an on-site questionnaire. The questionnaire was developed in consultation with experts and finalized through a pilot survey.

This study eliminated questionnaires with significant mistakes, such as duplicate and unreliable questionnaires with obvious inaccuracies in the respondents’ answers. Overall, 7430 valid questionnaires were collected. The current study’s questionnaire is readily available in Appendix 1 of the supplementary file. Additionally, Table S1 in the supplementary file presents the groupings, assignments, and definitions of the variables used in this study.

### Quality assurance

A team of investigators comprising staff from the CDC, township health centers, and the community conducted the questionnaire survey. Our research group provided comprehensive training for investigators on survey respondent selection and questionnaire content to ensure the accuracy of the results. The survey was conducted in households, and an on-site review of the questionnaires was performed to ensure completeness and logic. After data collection, two personnel carried out data entry to ensure accuracy. Personnel were compensated for their work on questionnaire collection and entry. Our research group organized and censored the entire data collection and entry process.

### Statistical analysis

EpiData (version 3.1, USA) was used to collect data and create a database. Descriptive analyzes was performed using IBM SPSS software (version 20.0, USA) to summarize the principal results.

Traditional regression analysis models, such as multiple linear, logistic, and Cox proportional risk regression models, may struggle when predicting complex social issues due to multicollinearity and high data dimensionality [22]. The commonly used coefficient compression method of ridge regression can offer high computational accuracy but cannot compress the coefficients to zero, thus retaining all variables in the equation, making it harder to explain the model [23]. In 1996, statisticians proposed a new coefficient compression method called LASSO (Least Absolute Shrinkage and Selection Operator) that effectively addresses the shortcomings of previous methods [24]. By introducing a penalty term (λ) to the model estimation, LASSO can effectively reduce regression coefficients for unnecessary variables to zero, ultimately removing them from the model and streamlining variable screening [25]. An appropriate value of λ can compress some of the regression coefficients to minimize the sum of squares of the residuals, and even some regression coefficients could be reduced to zero, thus eliminating them from the model [24].

A lasso-linear regression analysis was performed using SPSS and Stata 17.0 software. LASSO regression was used to exclude collinear variables and statistically non-significant. These screen variables considerably affected community health service utilization and ranked the coefficients of variables to obtain factors initially. Multivariate linear stepwise regression was then employed to select the factors influencing health service utilization. Finally, the regression model can achieve strong and concise explanatory power. *p* < 0.05 was statistically significant.

## Results

As summarized in Table 1, there were 3859 women and 3571 men, accounting for 51.9% and 48.1% of the respondents, respectively. The average age of respondents was 47.28 ± 16.51 years, 46.86 ± 16.53 years for men, and 47.67 ± 16.48 years for women, with a relatively even distribution by the age group. Overall, the educational level is relatively poor, and only 2.7% (201) of the respondents had an undergraduate or higher level of education. Approximately 43.8% of the respondents had an average monthly income per capita of ≥ 1000 and < 3000 Yuan, and 32.8% had ≥ 3000 and < 5000 Yuan. Most of the population were covered by at least one type of health insurance.

Specifically, there was a high coverage of officially organized basic medical insurance, with 46.4% of residents participating in the new rural cooperative medical scheme, 39.2% involved in the urban resident’s basic health insurance, and 13.2% in basic medical insurance for urban employees. Additionally, approximately 1.5% of residents purchased commercial medical insurance, and 0.4% enjoyed free medical care; 75.4% of residents reported spending less than 10% of their income on health services annually. Overall, 3202 (43.2%) residents had a family history of hypertension.

**Table 1:** Characteristics of Respondents

Variables	Grouping	Frequency	Valid Percentage (%)
Age (years)	< 20	286	3.8
	20–34	1566	21.1
	35–49	2315	31.2
	50–64	2021	27.2
	≥ 65	1242	16.7
Sex	Men	3571	48.1
	Women	3859	51.9
Education	No formal school education	564	7.6
	Did not finish primary school	928	12.6
	Primary school	1412	19.1
	Junior high school	2739	37.1
	Senior high school and technical secondary school	1153	15.6
	Junior college	384	5.2
	Undergraduates and above	201	2.7
The monthly average income per capita (Yuan)	< 500	288	4.3
	≥ 500 and < 1000	552	8.2
	≥ 1000 and < 3000	2950	43.8
	≥ 3000 and < 5000	2207	32.8
	≥ 5000	736	10.9
Medical Insurance*	Basic medical insurance for urban employees	965	13.2
	Free medical care	32	0.4
	Urban residents’ basic health insurance	2875	39.2
	New rural cooperative medical scheme	3403	46.4
	Commercial medical insurance	111	1.5
The percentage of annual income spent on health care	< 10%	5532	75.4
	≥ 10% and < 30%	1458	19.9
	≥ 30% and < 50%	184	2.5
	≥ 50% and < 70%	60	0.8
	≥ 70% and < 90%	31	0.5
	≥ 90%	68	0.9
Family history	Yes	3203	43.2
	No	4207	56.8

Note: *Multiple Options

### Chronic diseases

**Fig. 1:**
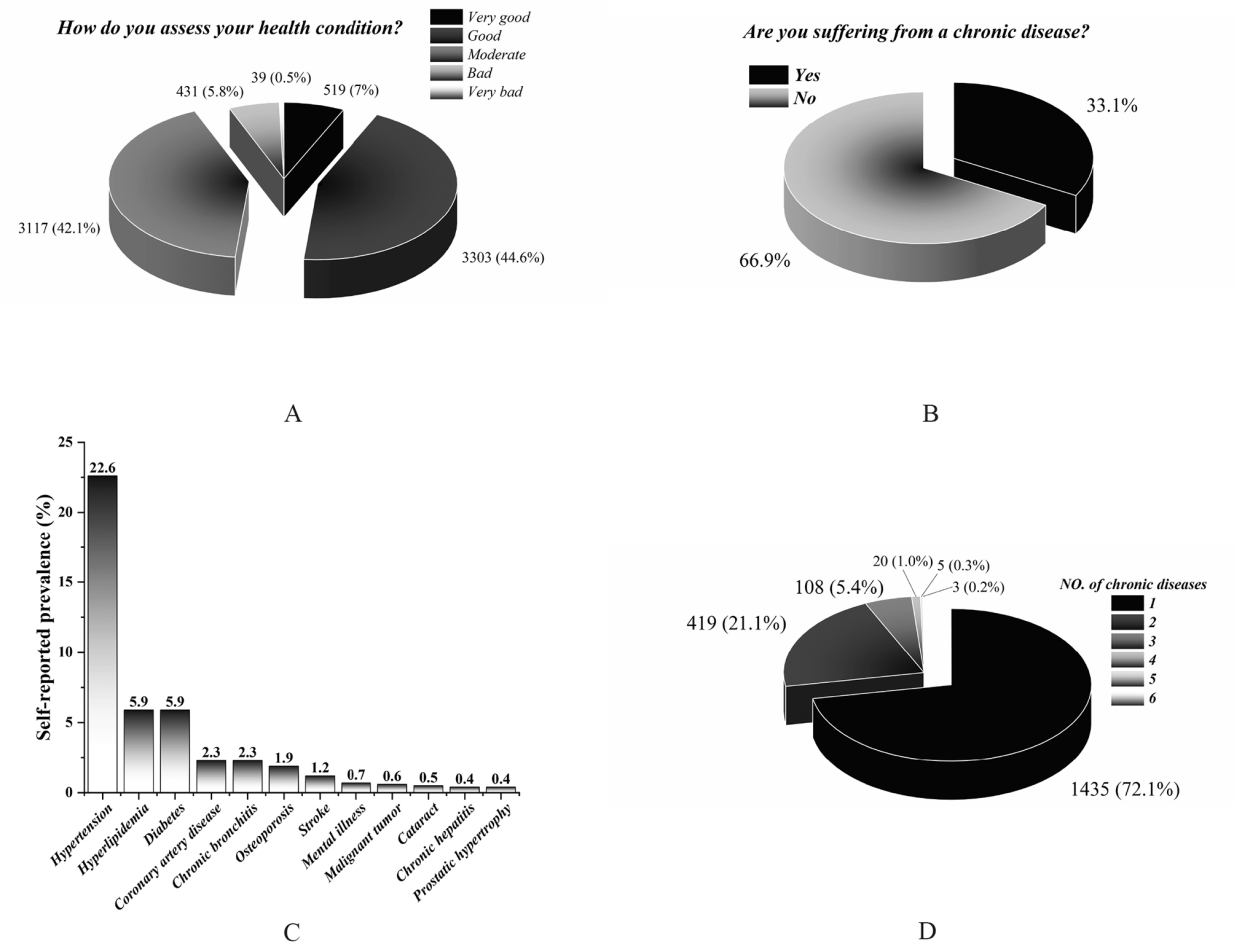
Chronic diseases. (**A**) self-reported health condition; (**B**) Are you suffering from a chronic disease; (**C**) Self-reported chronic diseases; (**D**) Number of chronic diseases

As shown in Fig. 1, most residents reported that their health was relatively good (44.6%) or moderate (42.1%), with 5.8% of residents considering their health bad (Fig. 1A). Chronic diseases affect 66.9% of the respondents (Fig. 1B). The most prevalent self-reported chronic diseases were hypertension (22.6%), hyperlipidemia (5.9%), diabetes (5.9%), coronary artery disease (2.3%), chronic bronchitis (2.3%), osteoporosis (1.9%), and stroke (1.2%), as seen in the Fig. 1C). Among the residents with chronic diseases, 72.1% had one of the aforementioned chronic diseases, 21.1%, 5.4%, 1.0%, 0.3%, and 0.2% had two, three, four, five, and six, respectively (Fig. 1D).

### Community health services

**Fig. 2:**
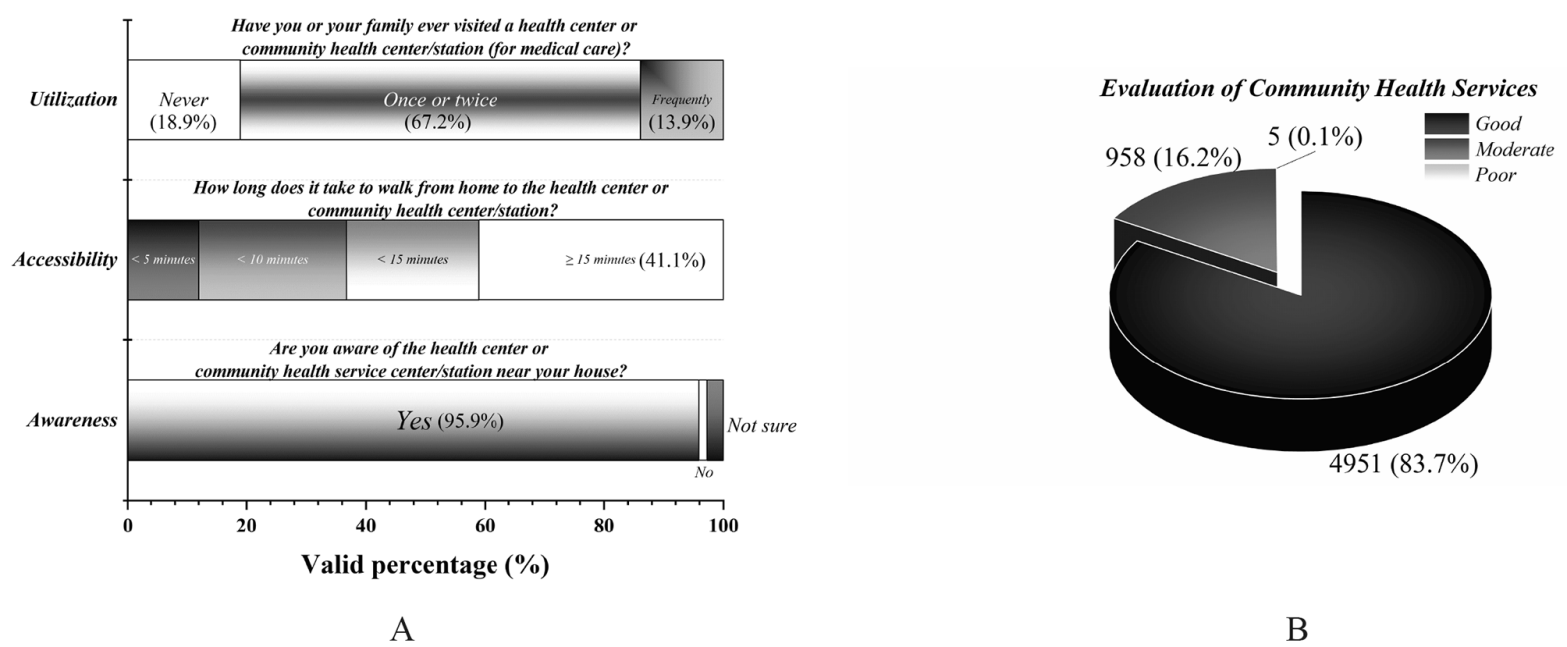
Community health services

As shown in Fig. 2A, the awareness rate of residents regarding community health service facilities was 95.9% (*Question: are you aware of the health center or community health service center/ station near your home?*). The survey results on the accessibility of health services showed that 41.1% of residents reportedly spent more than 15 min walking to a health service provider (*Question: how long does it take to walk from home to the health center or community health service center/ station?*). However, to collect information on health service utilization, the study asked the participants, “*have you ever visited a health center or community health service center/station for medical care?*” The results showed that 13.9% of residents visited frequently, 67.2% visited only once or twice, and 18.9% never visited.

The community health services offered were rated good by 83.7% of the respondents, while 16.2% and only 0.1% rated them as moderate and poor, respectively (Fig. 2B). Furthermore, of the residents who reported having chronic diseases, 14.1% of the respondents had never attended community health service.

### Lasso-linear regression

The dependent variable was community health service utilization, which took the values of 1, 2, and 3 as discrete quantitative data, and linear regression was used to analyze its principal influencing factors. The analysis was categorized into two parts: (1) considering its relatively multiple factors and possible collinearity concerns, LASSO regression was initially employed to select the main variables; (2) the selected independent variables were included in the linear regression analysis to analyze the determinants of health service utilization.

**Fig. 3:**
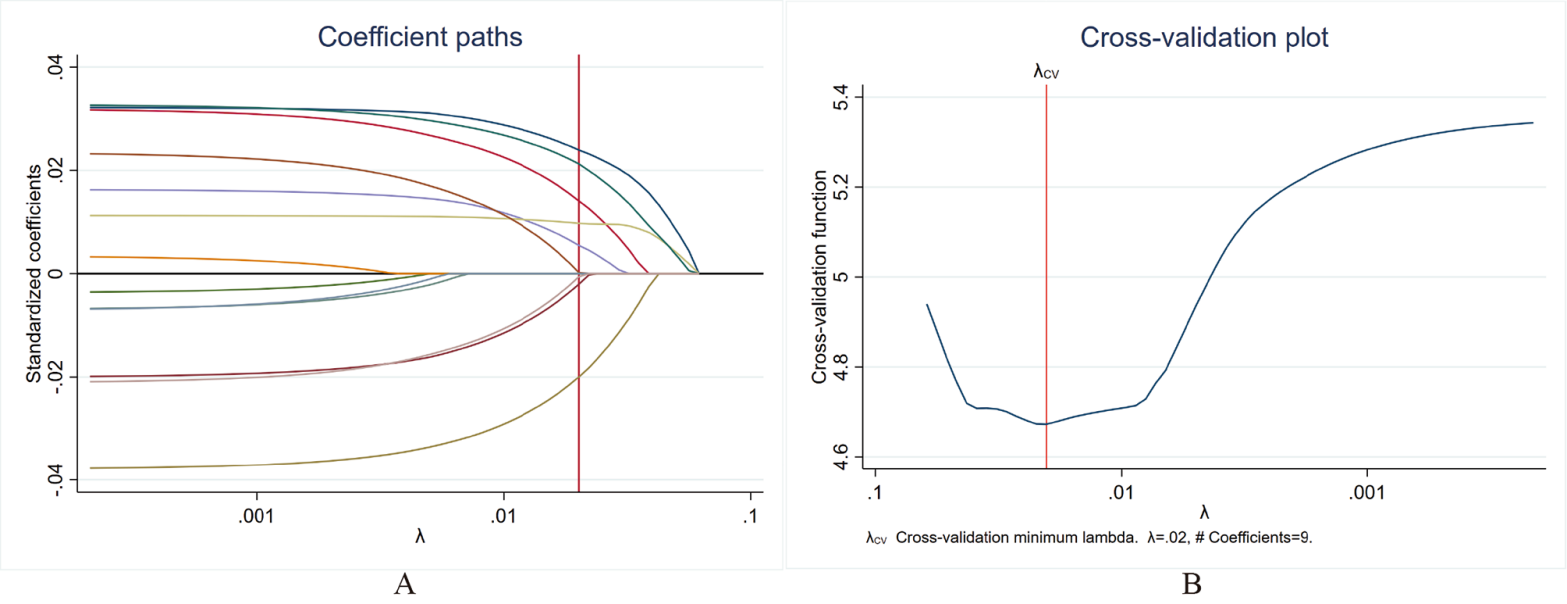
LASSO regression. (**A**) LASSO coefficient paths plot; (**B**) 10-fold cross-validation plot

Supplementary Table 1 (Table S1) of the supplementary files listed 13 potential influencing factors in the Lasso regression for variable selection. The Lasso coefficient paths plot (Fig. 3A) and 10-fold cross-validation (CV) plot (Fig. 3B) was constructed, and the seed number was set to 123,456. In the coefficient paths plot, the coefficient of variables was suppressed to zero under the penalty coefficient λ continuously grows. In the cross-validation plot, the red line marks the value of the best λ (λ = 0.0202) after Lasso 10-fold cross-validation, when the CV mean prediction error was smallest. Finally, nine variables were selected and ranked by variable coefficients as follows: age (0.0239), number of chronic diseases (0.0213), satisfaction with community health services (-0.0200), family history (0.0141), chronic disease or not (0.0098), self-reported health conditions (0.0055), sex (-0.0020), accessibility to community health services (-0.0006), and knowledge of chronic diseases (0.0002).

The results of the linear regression are generally consistent with those of the LASSO regression. As presented in Table 2, eight variables, namely, satisfaction with community health service (β=-0.098, *p* < 0.001), family history (*β* = 0.090, *p* < 0.001), Age ((*β* = 0.071, *p* < 0.001)), the number of Chronic diseases (*β* = 0.095, *p* < 0.001), self-reported health condition (*β* = 0.067, *p* < 0.001), knowledge of chronic diseases (*β* = 0.046, *p* < 0.001), the accessibility of community health service (*β*=-0.045, *p* < 0.001), and sex (*β*=-0.039, *p* < 0.001) were identified as significantly contributing factors to health service utilization. Specifically, the female residents, the higher the satisfaction with community health services, the higher the family history of chronic diseases, the older the person, the greater the number of chronic diseases, the worse the self-reported health condition, the richer the knowledge about chronic diseases, the better the accessibility to community health services, and the greater the health service utilization.

**Fig. 4:**
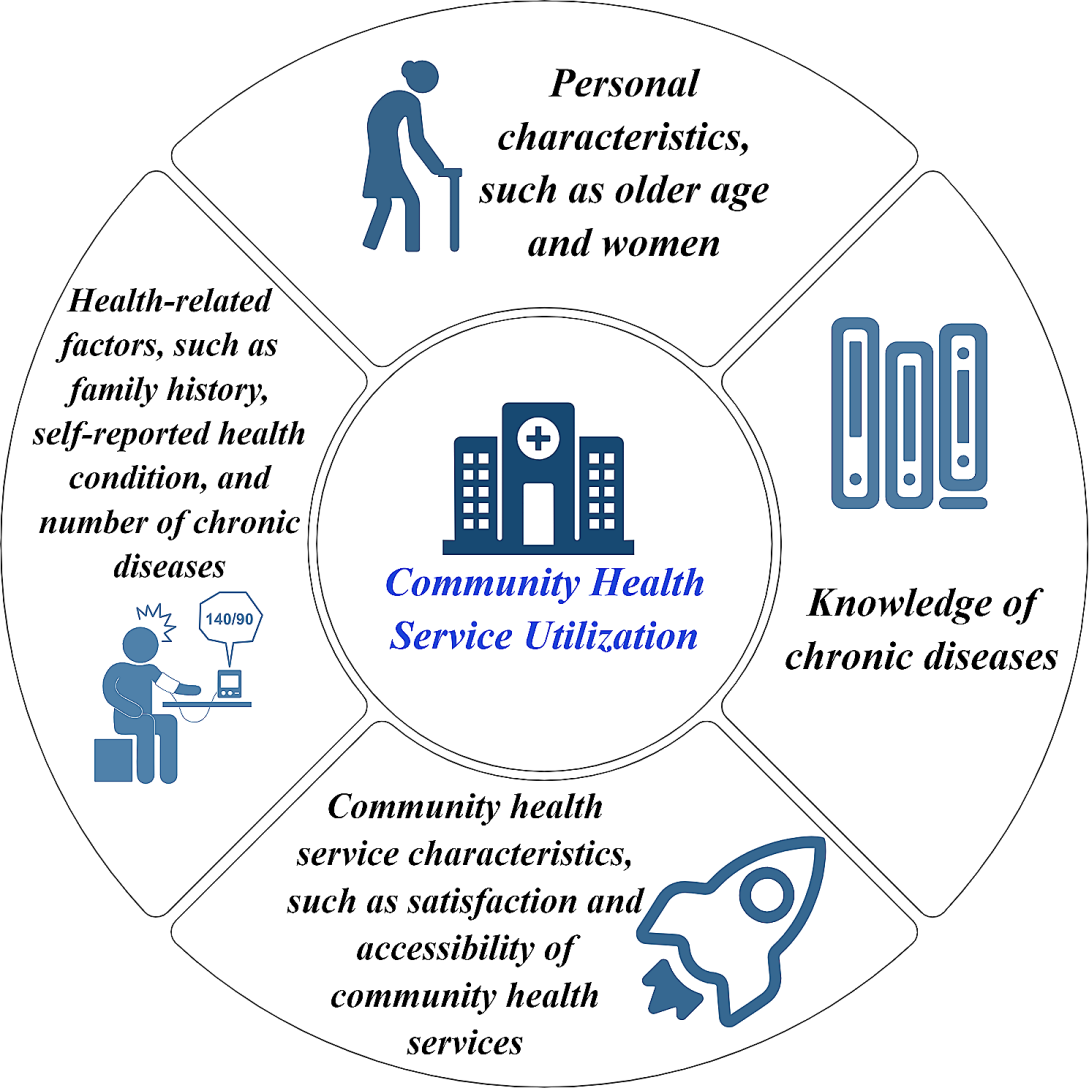
Determinants of community health services utilization

It can be simplified into four categories of determinants (Fig. 4): (1) personal characteristics, age, and sex; (2) health-related factors, such as family history, self-reported health condition, and the number of chronic diseases; (4) community health service characteristics, such as satisfaction with and accessibility to community health services; and (4) knowledge of chronic diseases.

**Table 2:** Linear regression results

Variables	B	Standardized Coefficients(β)	t	*p*	95% CI for B	
					Lower	Upper
Constant	2.125		58.072	< 0.001	2.053	2.196
The satisfaction of community health service	-0.102	-0.098	-6.643	< 0.001	-0.132	-0.072
Family history	0.075	0.090	5.990	< 0.001	0.051	0.100
Age (years)	0.026	0.071	4.248	< 0.001	0.014	0.038
NO. of Chronic disease	0.050	0.095	5.691	< 0.001	0.033	0.067
Self-reported health condition	0.035	0.067	4.059	< 0.001	0.018	0.052
Knowledge of chronic diseases	0.011	0.046	3.048	< 0.01	0.004	0.019
Accessibility of community health service	-0.017	-0.045	-3.034	< 0.01	-0.028	-0.006
Sex	-0.031	-0.039	-2.672	< 0.01	-0.055	-0.008

Note: B, Unstandardized Coefficients; CI, Confidence Interval

## Discussion

Chronic diseases have emerged as major public health challenges in modern society [26, 27]. A community-based cross-sectional study was conducted using a large random sample to investigate the self-reported prevalence of common chronic diseases among adults in Ganzhou, a city in southern China. The results showed that 66.9% of the residents had chronic diseases. This data is even higher than other studies reporting the prevalence of chronic diseases in the middle-aged and elderly (44.27%) [28]. The prevalence of chronic diseases would only be higher, considering undetected cases. Most residents reported that their health was relatively good (44.6%) or moderate (42.1%). The top three prevalences of self-reported chronic diseases were hypertension (22.6%), hyperlipidemia (5.9%), and diabetes (5.9%). This disease ranking is similar to the findings of the elderly in a place in Zhejiang Province, China, in which the top three chronic disease prevalence rates were hypertension (40.2%), dyslipidemia (8.4%), and diabetes (7%) [29]. Thus, the high prevalence of chronic diseases in the region is a severe threat to the health of residents, and common chronic diseases such as hypertension, hyperlipidemia, and diabetes should be priorities for primary care services.

In this study, 95.9% of the respondents were aware of the local community health facilities, indicating that community health services have been widely promoted. There is still work to be done to improve the accessibility of health services, especially in rural areas, since 41.1% of residents reported walking more than 15 min to a health service provider. Additionally, of the residents who reported having chronic diseases, 14.1% of the respondents had never attended community health services, indicating that the utilization of community health service resources needs to be enhanced. A systematic analysis revealed that providing community health services to rural elderly residents had improved remarkably in mainland China; however, multiple factors, from the individual to the system level, resulted in low access and utilization [30].

Eight factors clustered into four categories of determinants were included: personal characteristics, health-related factors, community health service characteristics, and knowledge of chronic diseases.

The results showed that personal factors such as age and sex were associated with residents’ health service utilization. As age grows, the greater the need for health services, the greater the utilization of health services. This result is comparable to those of similar previous studies [31], in which patients’ choice of community health services for their first treatment was influenced by age, with the elderly being more likely to prefer community health services. Interestingly, we found that women were more likely to use health services than men. One possible explanation for this is that women have a stronger perception of the risk of disease that drives access to health services. Gender is crucial in shaping risk perceptions and coping strategies, reflecting the predisposition of the public to accept health interventions and take precautionary measures [32]. The study found that when faced with risks such as the COVID-19 crisis, women’s perception of risk was higher and more in line with government guidelines [32], and this point should be applied to health risks such as chronic diseases.

Health-related factors are closely associated with health service utilization. Our study found that residents with higher health risks–specifically, a family history of the disease, poor self-perceived health, and a greater number of chronic conditions–were more likely to seek health services. This finding is consistent with the results of previous studies, which revealed that perceived health status was significantly associated with the use of health services [33] and that respondents with chronic conditions were more likely to use community health services than their counterparts [34]. Among urban residents aged ≥ 45 years, disease condition counts were positively correlated with outpatient or inpatient service use [35]. This is easily understood as health service utilization will only occur if there is a health service need, especially when arising from the disease itself.

The evaluation of health services is an important consideration for patients in selecting health services [31]. The level of community health services here, measured by the level of satisfaction and accessibility of health services to residents, determines whether residents would use health services. Studies have shown that travel time to the nearest Health Facility is significantly associated with health service utilization, and promoting access to health services at an affordable cost will possibly result in better utilization of healthcare services [33]. A study of medical care revealed that satisfaction was a one-way explanatory factor for utilization [36]. Collectively, community health-service providers can facilitate health-service accessibility while improving residents’ satisfaction with health services to drive health-service utilization.

The role of residents’ disease knowledge should not be ignored, as we aimed to improve the efficiency of the utilization of health services. As noted in an online cross-sectional study, knowledge may correlate with the utilization of mental health services among Black adults [37]. Health service providers should focus on health education efforts to promote disease knowledge acquisition among residents, which, in turn, promotes health service utilization.

In summary, the threat of chronic diseases among adult residents of southern China has become a concern as society evolves, and hypertension, hyperlipidemia, and diabetes should be priorities for primary care services. Promoting the utilization of community health services is a fundamental strategic measure in the context of limited health service resources. To address the challenge of increasingly prevalent chronic diseases, relevant government departments should focus on the roles of personal characteristics, health-related factors, health service levels, and disease knowledge in community health service utilization to develop an efficient health service utilization model that fits the local situation. Specifically, the priority measures involve enhancing residents’ access to and satisfaction with community health services and raising awareness of chronic illnesses among older individuals with poor health status.

However, this study has some limitations. (1) In the present study, community health services (Y, outcome variable) evaluated the overall utilization of health services rather than only those related to non-communicable diseases (NCDs), which could introduce bias in the study. However, China’s community health services serve as the “gatekeeper,” primarily focusing on managing chronic diseases among residents. Therefore, our findings may have some minor biases, but they are insignificant; (2) Although a large random sample was collected, it was concentrated in a city in Southern China, and the cross-sectional study was relatively weaker in explaining causality than the cohort study, which will somewhat limit the generalization of the findings; (3) Health service utilization is a socio-disciplinary issue, and its determinants may not work individually but synergistically with multiple factors, which should be explored further in future research; (4) Part of the data was collected during the COVID-19 pandemic in 2020, potentially influencing the findings. Despite this, given that the research site was relatively unaffected by the pandemic and the meticulous approach to sampling and quality control, the findings presented in this paper are only slightly influenced by the COVID-19 pandemic.

### Electronic supplementary material

Below is the link to the electronic supplementary material.

Supplementary Material 1

## Data Availability

Data are available from the corresponding author upon reasonable request.

## Abbreviations

NCDs: Noncommunicable chronic diseases
CDC: Centers for Disease Control and Prevention
LASSO: Least absolute shrinkage and selection operator
CV: Cross-validation
