# Supplementary material for: Chronic diseases and determinants of community health services utilization among adult residents in southern China: a community-based cross-sectional study
Source: BMC Public Health. 2024 Mar 28;24:919. doi: 10.1186/s12889-024-18435-8 (PMC10979594; doi:10.1186/s12889-024-18435-8)
Supplement: Supplementary file 1 — Supplementary Material 1 [file 12889_2024_18435_MOESM1_ESM.pdf]

Table S1 Groupings, assignments and definitions of variables

Variables	Grouping and assignments	Definition of variables
Age (years)	1="<20";2="20-34";3="35-49";4="50-64";5="≥65"	**X_1**
Sex	1="Men"; 0="Women"	**X_2**
Education	1="No formal school education";2="Did not finish primary school";3="Primary school";4="Junior high school";5="Senior high school and technical secondary school"; 6="Junior college"; 7="Undergraduate and above"	**X_3**
Whether to participate in a medical insurance?	1="yes";0="No"	**X_4**
The monthly average income per capita (Yuan)	1="<500"; 2="≥500 and <1000"; 3="≥1000 and <3000"; 4="≥3000 and <5000"; 5="≥5000"	**X_5**
Family history	1="yes";0="No"	**X_6:** *Do you have a family history of chronic disease?*
Self-reported health condition	1="Very good";2="Good"; 3="Moderate"; 4="Bad"; 5= "Very bad"	**X_7:** *How do you assess your health condition?*
Chronic disease	1="yes";0="No"	**X_8:** *Are you currently suffering from a chronic disease?*
NO. of Chronic disease	The higher the value, the more the number of chronic diseases.	**X_9**
Knowledge of chronic diseases	The higher the value, the higher the knowledge about chronic diseases.	**X_{10}:** *Which of the following groups are at high risk for chronic diseases?* There 5 types of risk groups were provided, and the identification of one is recorded as one point, followed by so on.
The awareness of community health service	1="Yes"; 2="Not sure"; 3="No"	**X_{11}:** *are you aware of the health center or community health service center/ station near you home?*
The accessibility of community health service	1="<5 min"; 2="<10 min"; 3="<15 min"; 4="≥15 min"	**X_{12}:** *how long it takes to walk from home to the health center or community health service center/ station?*

The satisfaction of community health service	1="Good"; 2="Moderate"; 3="Poor"	**X_{13}:** *How do you evaluate the services of the health center or community health service center/station in general?*
The utilization of community health services	1="Never"; 2="Once or twice"; 3="Frequently"	**Y:** *have you ever visited a health center or community health service center/ station for medical care?*

Appendix 1

Questionnaire

No.____________

I. Basic information

A1. Telephone number： ____________

A2. Date of Birth____________ (<yyyy/mm/dd>)

A3. Sex 1. Men 2. Women

A4. Ethnic 1. Han 2. Others

A5. What is your education level?

1. *No formal school education*
2. *Did not finish primary school*
3. *Primary school*
4. *Junior high school*
5. *Senior high school and technical secondary school*
6. *Junior college*
7. *Undergraduates and above*

A6. Your current marital status?

1. *unmarried*
2. *married*
3. *widowed*
4. *divorced*
5. *separated*
6. *others*

A7. Are you enrolled in medical Insurance?

1. *Enrolled*
2. *Not enrolled*
3. *Not sure*

A7a. What kind of medical insurance were you currently enrolled in?

1. *Basic medical insurance system for town staff*
2. *Public medical care*
3. *Urban Residents Basic Medical Insurance Institution*
4. *New rural cooperative medical system*
5. *Commercial medical insurance*
6. *Other ____________*

A8. What is your monthly average income per capita (Yuan)?

1. *less than 500*
2. *500 ~*
3. *1000 ~*
4. *3000 ~*
5. *5000 ~*

A9. Do you have a family history of chronic disease?

1. No 2. Yes

A9a. Have you experienced any of the following diseases in the past? (Multiple choice)

1. Hypertension
2. Diabetes mellitus
3. coronary heart disease
4. Malignant tumor
5. Stroke
6. Chronic bronchitis
7. Mental illness
8. Hyperlipidemia
9. Other ______________

A10. Percentage of money you spent on medical care this year?

1. less than 10%
2. 10%~30%
3. 30%~50%
4. 50%~70%
5. 70%~90%
6. >90%

II. Health status

B1. How do you assess your health condition?

1.Very good
2. Good
3. Moderate
4. Bad
5. Very bad

B2. Are you currently suffering from a chronic disease?

1. Yes 2. No

B2a. What diseases do you currently suffer from? (Multiple choice)

1. Hypertension
2. Coronary heart disease
3. Hyperlipidemia
4. Malignant tumor
5. Stroke
6. Diabetes
7. Mental illness
8. Osteoporosis
9. Chronic bronchitis
10. Chronic hepatitis
11. Cataract
12. Prostatic hypertrophy
13. Other diseases ____________

B3. Have you visited a health care facility in the last two weeks due to ill health?

1. Yes 2. No

B4. Have you been hospitalized in a medical facility in the past 12 months due to ill health?

1. Yes 2. No

B5. Have you been in a situation where you needed to be hospitalized in the last year, but you were not?

1. Yes 2. No

B5a. What was the reason you did not go to the hospital?

1. No need
2. No time
3. financial difficulty
4. Poor hospital service
5. Unavailability of medical beds
6. Self-administered traditional therapies
7. Untreatable by current medical technology
8. Other reason ____________

III. Knowledge of chronic diseases

C1. Which of the following are criteria for a high-risk group for hypertension? (Multiple choice)

1. Age ≥ 55 years
2. Family history of hypertension
3. Hyperlipidemia
4. Diabetes mellitus
5. Smoking
6.Overweight

C2. What are the possible complications that can arise from hypertension? (Multiple choices)

1.Stroke
2. Myocardial infarction
3. Kidney disease
4. Retinopathy

C3. What are the criteria for identifying a person who is at high risk for diabetes? (Multiple choices)

1. Age ≥ 40 years
2. Family history of diabetes
3. Hyperlipidemia
4. High blood pressure
5. Obesity
6. Overweight

C4. Which of the following groups are at high risk for chronic diseases? (Multiple choices)

1. Blood pressure level of 130~139/85~89mmHg (borderline hypertension)
2. Current smokers
3. Fasting blood glucose level of 6.1 ~ 7.0 mmol/L (Impaired fasting glucose)
4. Serum total cholesterol level of 5.2 ~ 6.2mmol/L
5. Men with waist circumference ≥ 90cm, women with waist circumference ≥ 85cm (central obesity)

IV. Community health services

D1. are you aware of the health center or community health service center/ station near you home?

1. Aware of

2. Not sure

3. Never heard of

D2.how long it takes to walk from home to the health center or community health service center/ station?

1.<5 min

2. <10 min

3. <15 min

4. ≥15 min

D3. How do you evaluate the services of the health center or community health service center/station in general?

1. Good

2. Moderate

3. Poor

D4. have you ever visited a health center or community health service center/ station for medical care?

1. Never

2. Once or twice

3. Frequently

D4. What is the main reason why you choose community health services for your medical care? (Multiple choice)

1. Convenient

2. Inexpensive

3. Possible reimbursement

4. Excellent standard of doctors

5. Good doctor's attitude

6. Can solve minor illnesses

7. Good equipment

8. Getting vaccinations

9.Listen to health education lectures

10. Free health checkups

11. Counseling health knowledge

12. Other ____________
